# Major Causes of Conflicting Interpretations of Variant Pathogenicity in Rare Disease: A Systematic Analysis

**DOI:** 10.3390/jpm14080864

**Published:** 2024-08-15

**Authors:** Tatyana E. Lazareva, Yury A. Barbitoff, Yulia A. Nasykhova, Andrey S. Glotov

**Affiliations:** 1 Department of Genomic Medicine, D.O. Ott Research Institute of Obstetrics, Gynaecology, and Reproductology, Mendeleevskaya Line 3, 199034 St. Petersburg, Russia; 2 Bioinformatics Institute, Kantemirovskaya St. 2A, 197342 St. Petersburg, Russia

**Keywords:** genetic variants, variant interpretation, conflicting interpretations of pathogenicity, ClinVar

## Abstract

The identification of the genetic causes of inherited disorders from next-generation sequencing (NGS) data remains a complicated process, in particular due to challenges in interpretation of the vast amount of generated data and hundreds of candidate variants identified. Inconsistencies in variant classification, where genetic centers classify the same variant differently, can hinder accurate diagnoses for rare diseases. Publicly available databases that collect data on human genetic variations and their association with diseases provide ample opportunities to discover conflicts in variant interpretation worldwide. In this study, we explored patterns of variant classification discrepancies using data from ClinVar, a public archive of variant interpretations. We found that 5.7% of variants have conflicting interpretations (COIs) reported, and the vast majority of interpretation conflicts arise for variants of uncertain significance (VUS). As many as 78% of clinically relevant genes harbor variants with COIs, and genes with high COI rates tended to have more exons and longer transcripts, with a greater proportion of genes linked to several distinct conditions. The enrichment analysis of COI-enriched genes revealed that the products of these genes are involved in cardiac disorders, muscle development, and function. To improve diagnoses, we believe that specific variant interpretation rules could be developed for such genes. Additionally, our findings underscore the need for the publication of variant pathogenicity evidence and the importance of considering every variant as VUS unless proven otherwise.

## 1. Introduction

The utilization of data from next-generation sequencing (NGS) has revolutionized medical genetics and genomics. With the increasing adoption of whole exome sequencing (WES) and whole genome sequencing (WGS), new challenges have been raised due to the fact that the large volume of data generated by NGS can be overwhelming. Due to the necessity of sifting through this vast amount of information to identify the specific variants truly responsible for a patient’s unique clinical presentation, bioinformatic analysis and interpretation have emerged as critical steps for accurate diagnosis. To streamline the variant interpretation process, the American College of Medical Genetics and Genomics (ACMG) has developed a standardized classification system. This classification categorizes genetic variants into five classes based on the strength of evidence supporting their association with diseases. These classes include pathogenic (P) (disease-causing), likely pathogenic (LP), variant of uncertain significance (VUS), likely benign (LB), and benign (B) [1]. The bioinformatic analysis of NGS data in medical genetics focuses on the comprehensive identification of the vast number of genetic variants present in each individual. Variant detection is followed by complex filtering and prioritization of the variants to identify the genuine cause of rare diseases. Prioritization is commonly done through the integration of variant features like the type, allele frequency (AF), and predicted consequence with the patient’s phenotype so as to identify the variants that most likely to contribute to the disease [2].

Despite the positive impact of the standardized ACMG classification framework and the development of sophisticated bioinformatic tools, significant challenges persist in interpreting genetic test results. One major obstacle lies in the limited number of patients with rare diseases. This makes it difficult to definitively classify and confirm the pathogenicity of rare variants, hindering our ability to identify mutations that could be targeted for therapeutic development [3]. Furthermore, the rapid adoption of NGS in clinical settings has outpaced our understanding of the relationships between genetic variations and specific phenotypes. This knowledge gap complicates pinpointing causal variants and contributes to the accumulation of VUSs. Additionally, discrepancies in variant classification arise between different laboratories and even between genetic laboratories and clinicians [4,5].

Biological factors contributing to a discordance of variant interpretations might include complex relationships between genotype and phenotype. Some genetic variants may not always cause disease (termed as low penetrance), and the severity of symptoms can vary greatly even among individuals with the same variant (termed as variable expressivity). Certain genetic diseases may not present symptoms until later in life or age of onset might vary, making it difficult to definitively link a variant to a specific clinical picture. A single genetic variant can sometimes lead to a wide range of phenotypes (a phenomenon termed phenotypic heterogeneity), further provoking different clinical interpretations of a single variant. The AF of disease-causing alleles might vary between different regions. For instance, cystic fibrosis (CF, OMIM #219700) is an autosomal recessive monogenic disorder caused by mutations in the cystic fibrosis transmembrane conductance regulator (*CFTR*) for which a significant diversity of pathogenic variants was found in patients of different ancestry [6,7,8,9]. Furthermore, studies have revealed ancestry-specific CF-causing variants. Notably, the F508del mutation, responsible for approximately 90% of CF cases in European populations, exhibits lower prevalence in other ancestries. In East Asians, for example, V520F is the most common variant, while G970D is particularly prevalent within the Chinese population [9,10]. These findings emphasize the critical role of collecting ethnicity-specific data and incorporating ancestry when interpreting genetic variants.

Technical limitations inherent to WES/WGS can contribute to variant classification inconsistencies. While WES/WGS has become widely used, the laboratory procedures remain complex and sensitive. The lack of fully standardized protocols can lead to inconsistencies, particularly in capturing challenging regions of the exome. These difficult-to-sequence regions, encompassing around 400 kbp for WGS and 1 Mbp for the best WES, include areas with low mappability due to features like pseudogenes, tandem repeats, and homopolymers [11,12]. The performance of variant callers and their sensitivity to adapter trimming can influence variant discovery [13]. Variant prioritization as a final step of bioinformatic analysis might also impact diagnosis through different prioritization strategies like phenotype-driven and network-driven approaches [14,15]. Some custom features might be implemented for variant scoring, such as local AF collection, and reports of solved cases available within the genetic center only. These can influence the WES/WGS clinical interpretation and contribute to discrepancies in variant classification. These discrepancies can have significant downstream consequences. Confusion may arise for both patients and healthcare providers, potentially leading to misinterpretations of genetic variants. For example, misinterpreting a variant associated with hereditary long QT syndrome could lead to unnecessary defibrillator implantation. This procedure, while potentially life-saving in some cases, can also have significant side effects [16,17].

A deeper understanding of the common factors that contribute to inconsistencies in clinical variant classifications is crucial for establishing greater confidence and standardization in clinical variant classifications. Publicly available databases, which aggregate and curate genetic variant information from various sources, are an excellent tool for this purpose. In our study we leverage a publicly available variant classification dataset from the NCBI ClinVar [18] resource to identify patterns and trends that contribute to genetic variant classification discrepancies. The analysis is aimed to identify both genetic variants and gene properties that are typical for cases of variant classification discordance.

## 2. Materials and Methods

### 2.1. Genetic Variant Data

ClinVar variant classification data were obtained from ClinVar VCF files between April 2018 and April 2024 (https://ftp.ncbi.nlm.nih.gov/pub/clinvar/) (accessed on 15 April 2024). The period was chosen according to the ClinVar website, which states that, before 2018, any discrepancy within the five ACMG/AMP pathogenicity terms was considered a conflict. Since 2018, conflicts are reported only for specific classifications (B/LB vs. VUS vs. P/LP). These changes could significantly impact our analysis. Furthermore, ClinVar did not provide data on the proportions of interpretations for variants with conflicting pathogenicity assessments prior to 2018. We further restricted the dataset to those genes annotated as having a robust association with rare diseases reported in OMIM (https://omim.org/). To assess the dynamic of conflicting interpretations of pathogenicity (COIs), records were subsequently filtered to include only those that were annotated with the status of COIs at least once up to April 2024 (a “conflicting_interpretations_of_pathogenicity” value of the CLNSIG field was used to retrieve the variants). An initial clinical interpretation (i.e., the last interpretation before conflict emerged) was ascertained based on the CLNSIG field for each variant for the latest ClinVar release before the emergence of COIs. Heterogeneity of interpretations was assesed by collecting and parsing all submitted classifications reported in the CLNSIGCONF column. ClinVar VCF files were annotated using Ensembl Variant Effect Predictor (VEP) to obtain information on variants’ predicted consequence severity (IMPACT rating) and AF from the gnomAD genomes dataset [19].

### 2.2. Analysis of Genes for Enrichment of Genetic Variants with COIs

A high frequency of conflicting interpretations for variants in a particular gene indicates the presence of systematic errors in the ascertainment of variant–disease associations. Hence, the identification of genes in which the frequency of COIs is significantly higher compared to an average gene in the genome is a task of pivotal importance. To assess whether a gene has a statistically significant enrichment of genetic variants with COIs compared to the average number of such variants found across the genome, a hyper-geometric *p*-value (*P* (*X* = *k*) ~*Hypergeometric*(*N*, *K*, *n*)) was computed for each gene. The total number of variants in a set of clinically relevant genes (as defined above) was taken as the universe size *N*, with *K* representing the total number of COI variants. Then, the total number of ClinVar records in each gene was taken as a sample size (*n*), and *k* corresponded to the number of COI variants in this gene. To control the type I error of multiple comparisons, the Benjamini–Hochberg false discovery rate (FDR) *p*-value adjustment was used. Biological functions and phenotypic characteristics shared by genes with many associated COI records were analyzed using the Gene Ontology (GO) gene set dataset from clusterProfiler 4.12.0 package for R v. 4.3.1 (https://r-project.org) [20] and the msigdbr package v.7.5.1 for acquiring Human Phenotype Ontology (HPO) gene set dataset from the Molecular Signatures Database (MSigDB) for *Homo sapiens* [21].

To analyze the power of the enrichment testing, we calculated the expected *p*-value for all combinations of the total number of variants (*n*, between 1 and 1000) and the percentages of variants with COIs (between 0 and 50). As shown in Figure S7, the method is expected to detect a significant enrichment for a modest (>15%) percentage of COI variants when the total number of variants in a gene exceeds 250 (a condition that is met for the majority of genes tested). For genes with 1000 variants or above, a COI rate of more than 10% is sufficient to be considered significant.

### 2.3. Analysis of Functional Properties of Genes Bearing COI Variants

To investigate the evolutionary constraint of genes, we utilized Loss-of-function Observed/Expected Upper Fraction (LOEUF) scores retrieved from the gene-level summary statistics provided by the Genome Aggregation Database (gnomAD) (https://gnomad.broadinstitute.org/downloads#v2-constraint) (accessed on 17 April 2024) [22]. These scores were pre-ranked and subsequently categorized into ten bins (deciles), with 0 representing the most depleted in protein-truncating variants (pLoF) and thus the most evolutionarily constrained genes, and 9 signifying no pLoF depletion and hence minimal constraint.

For genes reported in ClinVar, we obtained a canonical transcript and its length including UTR regions from Ensembl BioMart (ensembl.org/biomart/martview/) [23]. The number of exons annotated for each canonical transcript was counted based on GENCODE v. 45 genome annotation (https://gencodegenes.org/human/release_45.html) (accessed on 20 April 2024) [24]. For the investigation of isoform expression, transcript-wise data from the Genotype Tissue Expression (GTEx) V8 project was obtained via the GTEx portal (https://gtexportal.org) (accessed on 20 April 2024). The median expression level for each gene was used to determine the number of expressed isoforms. A transcript was considered expressed if its Transcripts Per Million (TPM) value exceeded 5 in at least one tissue type.

Data about the number and mode of inheritance of a rare disease were collected from the Human Phenotype Ontology (HPO) [25,26]. The relationship between gene and disease was ascertained using the OMIM identifier based on data provided by Ensembl BioMart.

### 2.4. Statistical Analysis

All statistical tests were conducted using R v.4.3.1 (https://r-project.org/) (accessed on 12 April 2024). Data visualization employed the following R packages: ggplot2 v.3.5.0 (https://ggplot2.tidyverse.org/) (accessed on 12 April 2024), scales v.1.3.0 (https://scales.r-lib.org) (accessed on 12 April 2024), and cowplot v.1.1.2 (https://wilkelab.org/cowplot/) (accessed on 12 April 2024). For continuous variables, we used the unpaired Wilcoxon test. For categorical variables, a chi-squared test with the appropriate number of degrees of freedom was used. In all cases, a significance threshold of α= 0.05 was used for hypothesis testing.

## 3. Results

### 3.1. Common Properties of Variants with Conflicting Interpretations of Pathogenicity

**Figure 1:**
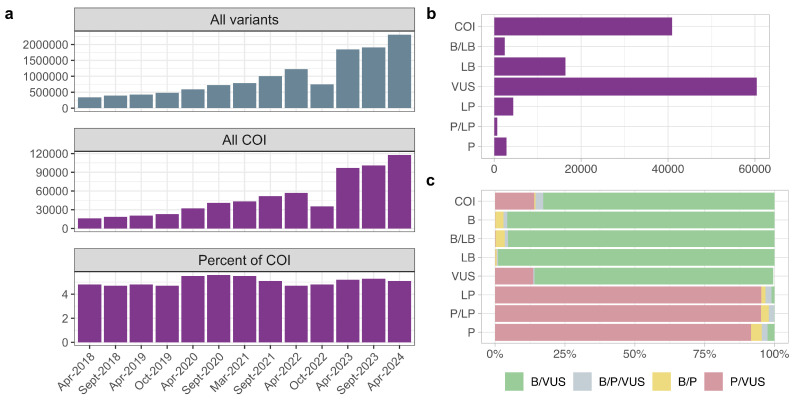
Dynamics of conflicting interpretations of variant pathogenicity. (**a**) A barplot showing the total number of ClinVar records (top), as well as the number (middle) and percentage (bottom) of variants marked as COI for the indicated releases of ClinVar data; (**b**) a barplot showing the number of COI variants with the indicated initial interpretation as of April 2018; (**c**) proportions of different combinations of submitted clinical significance records—B/VUS (variants with conflict between B/LB and VUS interpretation), P/VUS (variants with conflict between P/LP and VUS), B/P (conflict between P/LP and B/LB), and B/P/VUS (all three interpretations present).

The first goal of our work was to evaluate the overall prevalence of conflicting interpretations of variant pathogenicity in publicly available data. Hence, as a starting point of our analysis, we collected information about all genetic variants in genes associated with Mendelian disorders recorded in ClinVar between April 2018 and April 2024. In total, 2,296,245 variants in 4731 genes have been identified. Among them, 131,092 variants in 3703 genes had been assigned a COI tag at least once during the studied time period. A comparison of the number of COI variants as a function of time showed a steady increase in the number of such records, with an unexpected drop in both the total number of variants and COI variant count occurring in mid 2022 (Figure 1a). However, despite the rapid increase in the total number of variants recorded in ClinVar, the proportion of COI records did not change substantially over time, indicating that the accumulation of COIs did not outpace the overall rate of data generation (Figure 1a).

We next questioned what groups of variants tend to raise interpretation conflicts. To answer this question, following the initial collection of COI variants and the analysis of their accumulation rates, the variants were split based on the initial clinical significance interpretation (based on ACMG classification) before the emergence of the conflict. Interestingly, VUSs were the most common variants to enter into a conflict of interpretation, and the second-largest group of variants with COIs were initially classified as LB (Figure 1b). Notably, variants initially classified as pathogenic and likely pathogenic rarely entered into conflict (only 6.44% of all COI variants were initially interpreted as P/LP; see Figure 1b). We next questioned which combinations of interpretations are common for COI variants. To this end, we split all COIs into four major groups—B/VUS (including variants with a conflict between B/LB and VUS interpretation), P/VUS (variants with a conflict between P/LP and VUS), B/P (a conflict between P/LP and B/LB), and B/P/VUS (all three interpretations present). The conflicts commonly emerged between VUS vs. B/LB and VUS vs. P/LP groups (Figure 1c).

Next, we investigated the gnomAD data to see how the AF of genetic variations with COIs compared to the AF of variants from variants in different pathogenicity classes without conflicting interpretations. Both globally and when considering the maximum population frequency (popmax), the AF of COI variants was higher than observed for VUS, but lower than for B/LB variants. The distribution of the minimum AF (popmin, calculated across gnomAD ancestry groups) of variants with COIs is consistent with popmin AF of B/LB variations (see Figure S1).

The assessment of the predicted variant consequence severity (“IMPACT rating”) provided by Ensembl VEP corroborated previous observations. Thus, the proportions of variants classified as high-impact variants (e.g., frameshift variants, stop gained, splice-site variations) and moderate-impact variants (non-disruptive but potentially harmful, for example, inframe indels, missense variants) within the COI group were consistent with those observed in VUS and B/LB variants, but not with P/LP variants. The allele frequencies of variants with COI were thus consistent with the classes of variants that commonly entered into an interpretation conflict. Furthermore, when stratified by the initial interpretation before the COI designation, the distribution of predicted impact within the COI group closely resembled the classification categories before the emerged conflict. However, a greater prevalence of high- and moderate-impact variants was observed for COI variants that were initially classified as B/LB (compared to B/LB variants without conflicts). In contrast, for COI variants initially reported as VUS or P/LP, a greater proportion of milder variants was observed. These results suggest that interpretation conflicts are, at least in part, driven by the variant consequence at the protein level (Figure S2).

According to the ClinVar release from 7 April 2024, conflicts have been resolved for 13,528 of COI variants, which have been thus reclassified into one of the ACMG classification groups (Figure S3). The vast majority of such variants were reclassified into the B/LB (*n* = 4202 ) or LB (*n* = 3678) classes, in good concordance with the overall proportions of different interpretations for COI variants shown on Figure 1b,c.

### 3.2. Properties of Genes with a High Proportion of COI Variants

Having considered the properties of genetic variants that have conflicting interpretations of pathogenicity, we next asked if certain genes contain a higher proportion of such variants compared to an average gene in the genome. To test this hypothesis, we performed gene-based association analysis to identify genes harboring an enrichment of COI variants. Out of 3703 genes bearing at least 1 COI variant, as many as 285 (7.7%) genes displayed a statistically significant enrichment of COI variants (see the Methods section for a description of the enrichment testing procedure).

**Figure 2:**
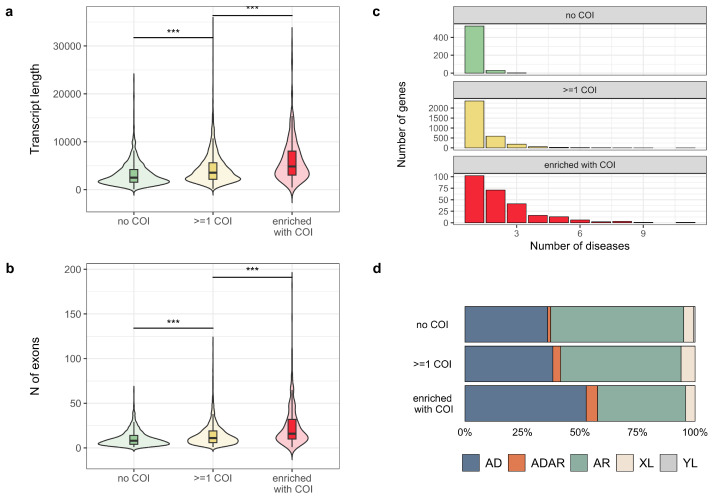
Common features of genes bearing COI variants. On all panels, no COI—genes without COI variants, ≥1 COI—genes bearing at least one COI variant, enriched with COI—genes displaying a significant enrichment with COI records. (**a**,**b**) Violin plots showing the canonical transcript length (**a**) or the number of exons (**b**) for different groups of genes (*** -*p*-value < 0.001); (**c**) histograms showing the number of associated disorders for indicated gene groups (no COI vs. ≥1 COI group: Wilcoxon test, *p*-value <2.2 × 10^−16^; ≥1 COI vs. genes enriched with COI: Wilcoxon test, *p*-value = 5.2 × 10^−9^); (**d**) barplots representing proportions of inheritance modes of disorders linked to genes from indicated groups (no COI vs. ≤1 COI group: chi-squared test, *p*-value = 3.78 × 10^−9^; ≥1 COI vs. genes enriched with COI: chi-squared test, *p*-value = 9.4 × 10^−13^.

To identify common features of genes with COI variants, we compared various gene-level properties, such as evolutionary constraint metrics, gene length, and structural complexity, between COI-enriched genes, genes with at least one COI, and genes lacking such variants. According to the results of this analysis, genes with COI variants are more constrained (Wilcoxon test, *p*-value = 7.4 × 10^−8^) (Figure S4) and exhibit significantly greater transcript length and number of exons (Wilcoxon test, *p*-value < 2.2 × 10^−16^) compared to genes with no reported COIs. Notably, COI-enriched genes had even longer canonical transcripts (Wilcoxon test, *p*-value = 6.1 × 10^−10^) and a higher number of exons (Wilcoxon test, *p*-value = 3.1 × 10^−12^) compared to genes without COI enrichment (Figure 2a,b). Furthermore, genes harboring COI variants displayed a significantly higher number of associated diseases compared to those lacking such variants (Wilcoxon test, *p*-value < 2.2 × 10^−16^). Similarly, COI-enriched genes had a higher number of associated diseases compared to non-enriched genes (Wilcoxon test, *p*-value < 5.2 × 10^−9^) (Figure 2c). Interestingly, the COI variant-bearing genes were also enriched for disorders with autosomal dominant inheritance (chi-squared test, *p*-value = 3.78 × 10^−9^). Again, the association between COI variants and autosomal dominant inheritance was further strengthened when considering only COI-enriched genes compared to non-enriched ones (chi-squared test, *p*-value = 9.4 × 10^−13^) (see Figure 2d). Finally, COI-enriched ones displayed a higher number of highly expressed isoforms (Wilcoxon test, *p*-value = 0.011) (Figure S5).

**Figure 3:**
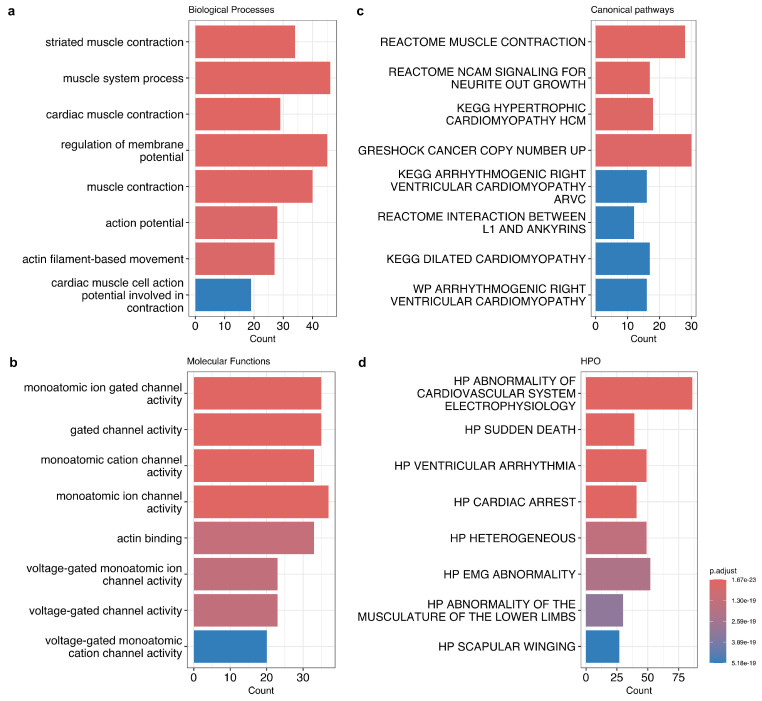
Barplots showing gene set enrichment analysis results for COI-enriched genes: (**a**) GO biological processes, (**b**) GO molecular functions, (**c**) canonical pathways, and (**d**) HPO gene sets from MSigDB. The color gradient represents the adjusted significance level.

To elucidate which functions are typical for genes with a high frequency of variant interpretation conflicts, we employed gene set enrichment analysis to investigate the biological processes regulated by genes enriched with such variants (see Figure 3a). This analysis revealed a striking enrichment for genes involved in muscle tissue morphogenesis and function, cardiac structures’ development, and heart contraction. Notably, genes essential for sensory organ development are also significantly overrepresented within the COI-enriched set.

We further delved into the molecular functions and canonical pathways associated with COI-enriched genes (Figure 3b,c). Interestingly, we observed a significant enrichment for genes with terms related to muscle contraction (e.g., *TTN* (12% of COIs; FDR-adjusted *p*-value = 1.48 × 10^−203^), *KCNJ11* (27% of COIs; FDR-adjusted *p*-value = 1.03 × 10^−43^), *DYSF* (11% of COIs; FDR-adjusted *p*-value = 1.02 × 10^−37^), *ACTN2* (14% of COIs; FDR-adjusted *p*-value = 5.48 × 10^−27^), *MYBPC3* (10% of COIs; FDR-adjusted *p*-value = 5.8 × 10^−24^), *NEB* (8% of COIs; FDR-adjusted *p*-value = 7.9 × 10^−23^)), including actin (e.g., *ACTN2*, *DMD* (7.8% of COIs; FDR-adjusted *p*-value = 2.3 × 10^−13^), *SYNE1* (9% of COIs; FDR-adjusted *p*-value = 3.35 × 10^−22^)), calmodulin binding (e.g., *CACNA1S* (10.6% of COIs; FDR-adjusted *p*-value = 1.2 × 10^−17^), *MYO3A* (9.5% of COIs; FDR-adjusted *p*-value = 6.4 × 10^−4^), *RYR2* (8% of COIs; FDR-adjusted *p*-value = 1.8 × 10^−17^), and *SCN5A* (10% of COIs; FDR-adjusted *p*-value = 1.1 × 10^−23^). Actin filaments are core components of the sarcomere, and calmodulin plays a crucial role in regulating muscle contraction through calcium signaling. Additionally, enrichment for genes involved in the transmembrane transfer of ions by voltage-gated channels was identified (e.g., *KCNE1* (12% of COIs; FDR-adjusted *p*-value = 1.5 × 10^−4^), *KCNE2* (16% of COIs; FDR-adjusted *p*-value = 2.4 × 10^−4^), *SCN4A* (8.9% of COIs; FDR-adjusted *p*-value = 3.2 × 10^−7^), and *SCN1B* (10% of COIs; FDR-adjusted *p*-value = 7 × 10^−4^)). These channels are essential for maintaining the electrical gradients necessary for muscle excitation–contraction coupling. Pathogenic variants of *KCNQ1* (9% of COIs; FDR-adjusted *p*-value = 2.2 × 10^−9^) and *KCNE1* that encode the subunits of potassium channels are causing factors of cardiac rhythm disturbance (OMIM #192500, #609621, #220400, #607554, #612347, #613695), which sometimes results in sudden death. A total of 25 genes (8.77%) from the 285 COI-enriched candidates were identified as having associations with various cardiomyopathy subtypes, including hypertrophic, dilated, and arrhythmogenic cardiomyopathy (e.g., *DSC2* (9% of COIs; FDR-adjusted *p*-value = 8.2 × 10^−6^), *DSG2* (10% of COIs; FDR-adjusted *p*-value = 1.6 × 10^−10^), *DSP* (15% of COIs; FDR-adjusted *p*-value = 9.2 × 10^−101^), *MYL2* (9.4% of COIs; FDR-adjusted *p*-value = 8.5 × 10^−3^), *PKP2* (8.7% of COIs; FDR-adjusted *p*-value = 1.6 × 10^−5^), and *JUP* (12% of COIs; FDR-adjusted *p*-value = 3 × 10^−13^)).

HPO terms’ enrichment analysis of genes with high COI rates corroborated the aforementioned findings, identifying 83 genes associated with abnormal cardiovascular electrophysiology. Furthermore, 39 genes were implicated in sudden death. Notably, the enriched terms primarily encompassed muscle weaknesses, amyotrophy, and abnormal cardiac function (Figure 3d). Collectively, these findings suggest that the cases of conflicting interpretations of variant pathogenicity might be overrepresented in genes associated with muscle and cardiovascular diseases.

Alternatively, genes with no reported COI are enriched in gene sets associated with germ cell development and implicated in infertility mainly caused by spermatogenic failure (e.g., *CATIP, CFAP65, CEP19*) and oocyte/zygote/embryo maturation arrest (e.g., *PADI6, REC114, WEE2*). These genes are also linked to mononuclear cell proliferation and abnormal bronchus physiology results, for instance to bronchoconstriction (Figure S6).

## 4. Discussion

The analysis of ClinVar, a public archive for variant interpretations, revealed over 2.2 million variants with about 5.7% of variants with COIs reported in the database. The data show a significant increase in the number of genetic variants identified in Mendelian disease genes over the past six years. Interestingly, the proportion of variants with COIs slightly decreased over time. This suggests that the field is rapidly generating new variant data, but the process of assigning clinical significance to variants is more consistent. The rise in variant classification consistency likely stems from a combination of factors. First, standardized guidelines from the ACMG and European societies of medical genetics have provided a foundational framework. Second, initiatives like ClinGen and Sherloc have further refined these guidelines by establishing additional rules and promoting their implementation. Despite advancements, hereditary cancer and cardiac disorders continue to exhibit high rates of variant interpretation discordance, particularly at the border between VUS and B/LB [27]. This aligns with our findings of discordance frequently arising between VUS and B/LB.

We found that the largest group of variants entering a conflict of interpretation are VUSs. VUSs are known to complicate the clinical decision in around 10% to 41% of cases being analyzed by multigene panels, leaving clinicians unsure about a patient’s disease risk, and making it difficult to recommend appropriate preventive measures or treatment [28]. VUSs may trigger additional unnecessary testing and potentially delay the diagnosis of confirmed conditions. The reevaluation of VUS status requires additional evidence, such as population-specific allele frequency, functional studies, or RNA-seq analysis. Establishing evidence-based guidelines for managing VUSs can help reduce disparate assessments by academics and doctors and provide consistent recommendations to patients. However, it is extremely important to emphasize that novel genetic variants should not be assigned to a class other than VUS without strong evidence of their benign/pathogenic nature [29]. Indeed, the automated ACMG criteria-based classification of variants with reported COIs indicates that for the vast majority (129,953 out of 131,092, 99.1%) of these variants there is no publicly available evidence to classify them as benign or pathogenic (Table S1).

Furthermore, we explored the properties of genes harboring variants with COI to identify factors associated with variants’ interpretation discordance. The study revealed that 78% of clinically relevant genes harbor variants with COIs. Interestingly, these genes exhibit a trend towards increased complexity compared to genes lacking reported discordance. This complexity manifests in two key aspects: clinically relevant genes with COIs tend to have longer canonical transcripts and more exons compared to ones without variant interpretation discordance. Complex gene structures often translate to a greater number of transcript isoforms. The presence of multiple isoforms potentially leads to a wider range of variant effects on gene products, further complicating the interpretation of variants’ clinical significance and being the possible source of interpretation discordance. In 2022, the Matched Annotation from NCBI and EMBL-EBI (MANE) transcript set was proposed, offering a standardized set of transcripts specifically designed for clinical genetics [30]. The widespread adoption of MANE has the potential to significantly reduce the discordance of interpretation associated with discrepancies arising from transcript annotation differences [2]. However, a recent study supposed that the underrepresentation of different ancestries in medical genetic studies also plays an important role in the instability of the pathogenicity classification of variants, as exemplified by a recent report for a set of 16 autosomal dominant cardiomyopathy-associated genes, over 7% of which undergo reclassification [31].

Next, we discovered 285 genes that are significantly enriched with variants with COI. These genes have a tendency to have more associated diseases per gene compared to non-COI-abundant ones. The enrichment analysis revealed the prevalence of a broad range of terms related to muscle development and function, from actin-binding to cardiac electrophysiology (Figure 3). Our findings align with previous studies on phenotypic heterogeneity (whereby individuals harboring pathogenic variants within the same gene can exhibit a spectrum of disease severity, or even develop entirely different disorders). Earlier studies by our group and other laboratories showed that phenotypic heterogeneity is common in genes linked to cardiovascular pathology [32,33,34,35]. Furthermore, the features of COI-enriched genes are strikingly similar to features of genes associated with multiple rare diseases, which are also enriched for constrained genes with multiple transcript isoforms and autosomal dominant inheritance [36]. Indeed, a high degree of phenotypic heterogeneity might explain the observed accumulation of COI variants within genes linked to multiple diseases, often exhibiting autosomal dominant inheritance, particularly those related to neuromuscular and cardiovascular pathologies.

While our study demonstrates several important trends of variant interpretation conflicts, there are several important limitations that have to be considered. First, our study was focused on gene–disease associations already reported in public databases such as OMIM and ClinVar. This limited our ability to discover novel gene–disease relationships. Second, we only retrieved data on variant interpretation conflicts from ClinVar, as a single public resource that contains variant classification data across the spectrum of inherited diseases. However, other publicly funded and commercial variant databases like COSMIC [37] containing somatic mutation data, or HGMD [38], a comprehensive collection of genetic variants with well-established pathogenicity, may be leveraged to provide more information for specific disease groups. It is also important to note that the lack of ethnicity data for each submitted record in ClinVar could contribute to false-positive emergencies of pathogenicity discrepancies. This is because population-specific genetic variations can influence variant interpretation. Finally, while most genes with over 15% of COI variants contained sufficient data for detecting statistically significant enrichment, a subset of genes exhibited relatively low variant counts, limiting our ability to identify significant COI-associated enrichment (Figure S7).

## 5. Conclusions

Overall, our study highlights the complex interplay between COI variants, gene characteristics, and disease phenotypes. The observed associations suggest that the phenotypic heterogeneity might contribute to the accumulation of discordance of variant interpretations and provide valuable insights for refining variant annotation and prioritization strategies, particularly in the context of genes with a high burden of COI variants and links to multiple diseases.

To further improve rare disease diagnosis, more attention could be paid to two key areas: variant interpretation and public database enrichment. Developing specialized rules for variant annotation and prioritization would be particularly impactful for genes linked to functions like cardiac conduction and muscular development and function. Expanding variant metadata in public genetic databases by including information like ethnicity and deeper phenotyping data would improve our ability to resolve conflicting interpretations of variants.

## Data Availability

All data and code pertinent to the analysis presented in this work are available at https://github.com/tanya-lazareva/coi.git (accessed on 15 July 2024).

## Supplementary Materials

The following supporting information can be downloaded at: https://www.mdpi.com/article/10.3390/jpm14080864/s1: Figure S1: AF of variants reported in ClinVar based on gnomAD v.2.1; Figure S2: IMPACT rating of all variants and COI variants splitted by initial interpretation; Figure S3: A barplot showing the number of variants for which COIs were reported with the indicated final interpretation as of April 2023; Figure S4: Genes associated with rare diseases, categorized by the presence of conflicting variants of interpretation—no conflicting evidence (no COIs), and at least one COI (≥1 COI), further stratified by LOEUF deciles; Figure S5: Histogram showing the distribution of the number of expressed transcripts (>5 TPM in at least one tissue according to the Genotype–Tissue Expression (GTEx) data) for COI-enriched genes (enriched with COIs) and genes with at least one COI and no enrichment (≥1 COI); Figure S6: Barplots showing gene set enrichment analysis results for no-COI genes; Figure S7: Heatmaps displaying hypergeometric test *p*-values. Table S1: Results of automated ACMG criteria-based classification of variants with reported COIs.

## Abbreviations

The following abbreviations are used in this manuscript:

COI	conflicting interpretations of pathogenicity
HPO	Human Phenotype Ontology
NGS	next-generation sequencing
OMIM	Online Mendelian Inheritance in Man
