# Supplementary material for: Major Causes of Conflicting Interpretations of Variant Pathogenicity in Rare Disease: A Systematic Analysis
Source: J Pers Med. 2024 Aug 15;14(8):864. doi: 10.3390/jpm14080864 (PMC11355203; doi:10.3390/jpm14080864)
Supplement: Supplementary file 1 [file jpm-14-00864-s001.zip › jpm-3099412-supplementary/Supplementary Figures S1-S7.pdf]

Major causes of conflicting interpretations of pathogenicity: a systematic analysis

Tatyana E. Lazareva[1], Yury A. Barbitoff[1,2*], Yulia A. Nasykhova[1], Andrey S. Glotov[1*]

[1] - Dpt. of Genomic Medicine, D.O. Ott Research Institute of Obstetrics, Gynaecology, and Reproductology, St. Petersburg, Russia

[2] - Bioinformatics Institute, Kantemirovskaya st. 2A, 197342 St. Petersburg, Russia

* - to whom correspondence should be addressed: barbitoff@bk.ru, anglotov@mail.ru

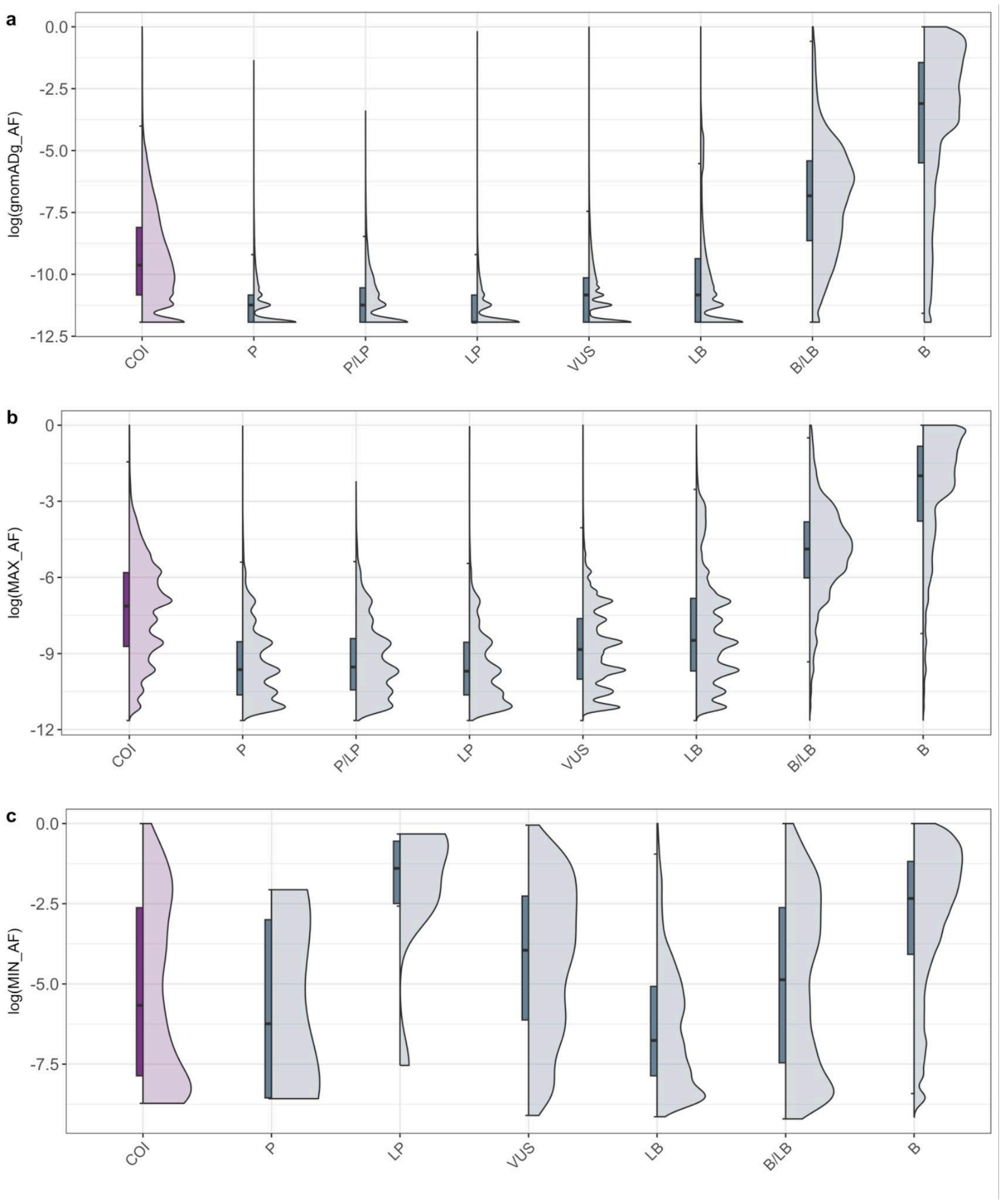

Supplementary Figure S1. AF based on gnomAD v.2.1 (a) global AF, (b) AF for the non-bottlenecked population with the highest (b) and lowest (c) frequency.

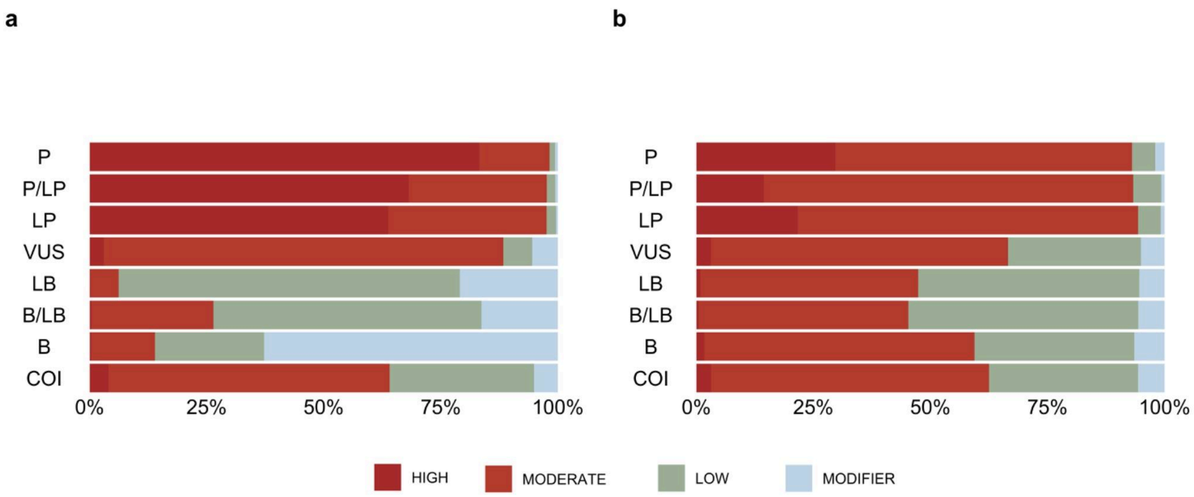

Supplementary Figure S2. IMPACT rating of (a) all variants, (b) COI variants splitted by initial interpretation (last record of ACMG classification before the conflict emerged).

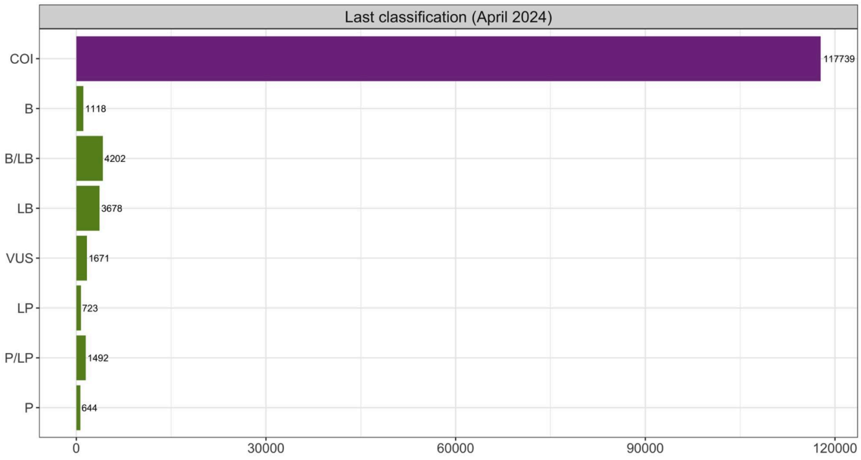

Supplementary Figure S3. A barplot showing the number of variants for which COI were reported with the indicated final interpretation as of April 2023

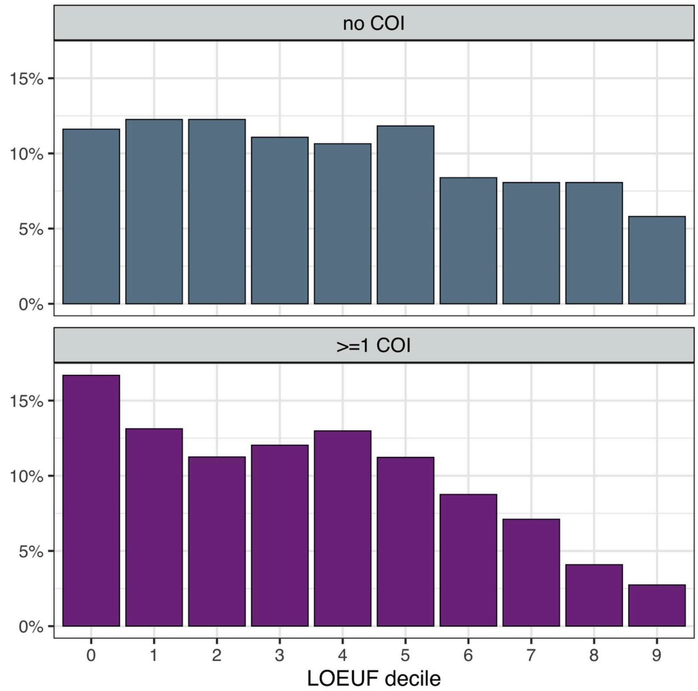

Supplementary Figure S4. Genes associated with rare diseases, categorized by presence of conflicting variants of interpretation - no conflicting evidence (**no COI**), and at least one COI (**>=1 COI**), further stratified by LOEUF deciles.

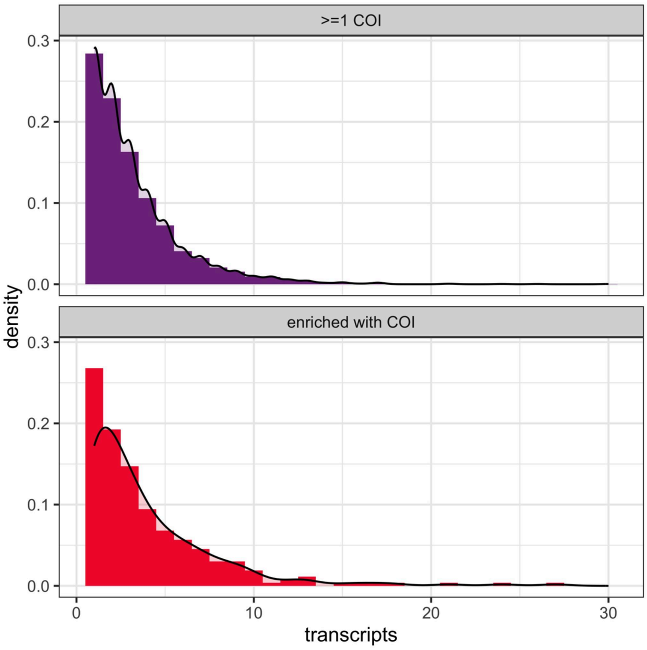

Supplementary Figure S5. Histogram showing the distribution of the number of expressed transcripts (>5 TPM in at least one tissue according to the Genotype-Tissue Expression (GTEx) data) for COI-enriched genes (**enriched with COI**) and genes with at least one COI and no enrichment (**>= 1 COI**).

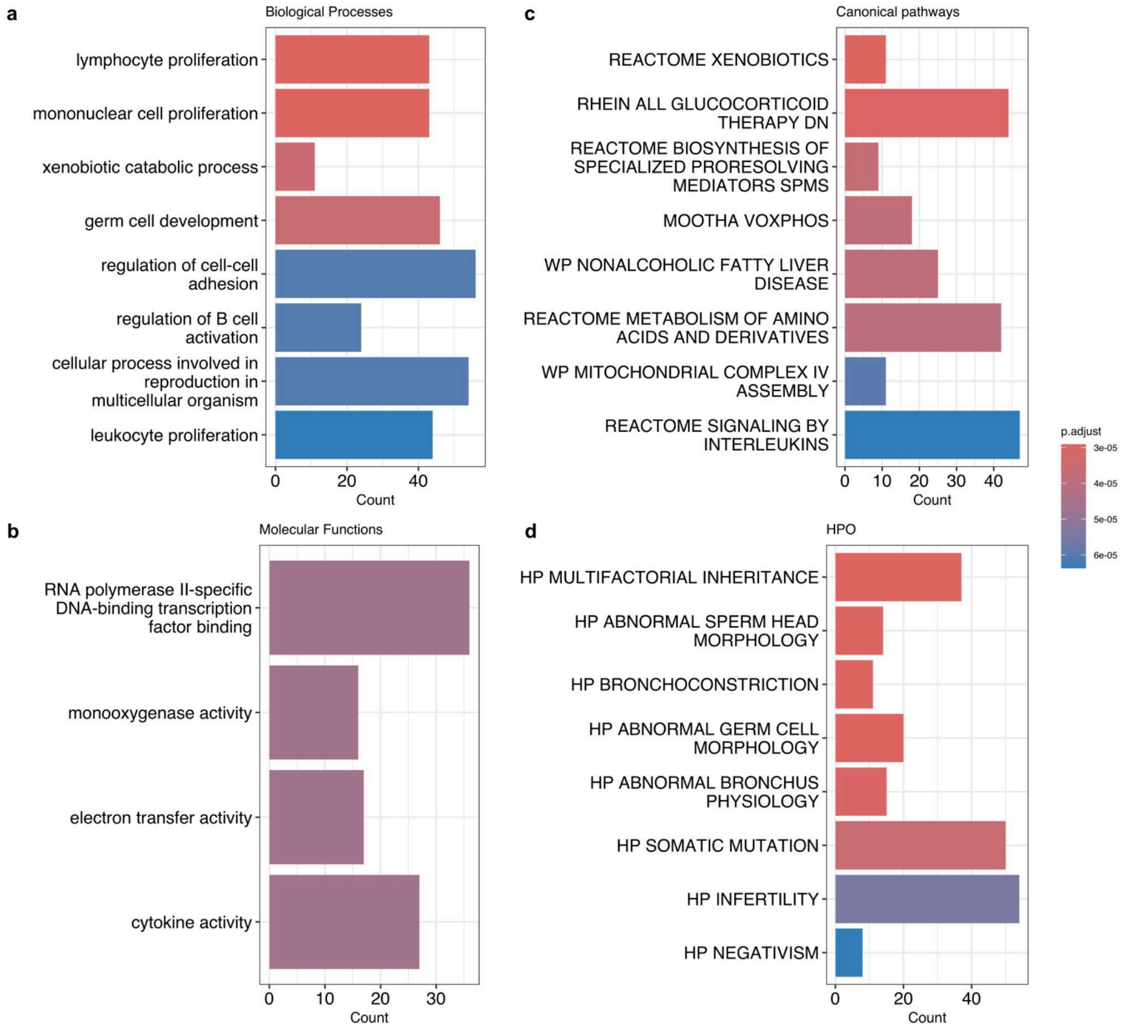

Supplementary Figure S6. Barplots showing gene set enrichment analysis results for no COI genes: (a) GO biological processes, (b) GO molecular functions, (c) canonical pathways, and (d) HPO gene sets from MSigDB. The color gradient represents the adjusted significance level.

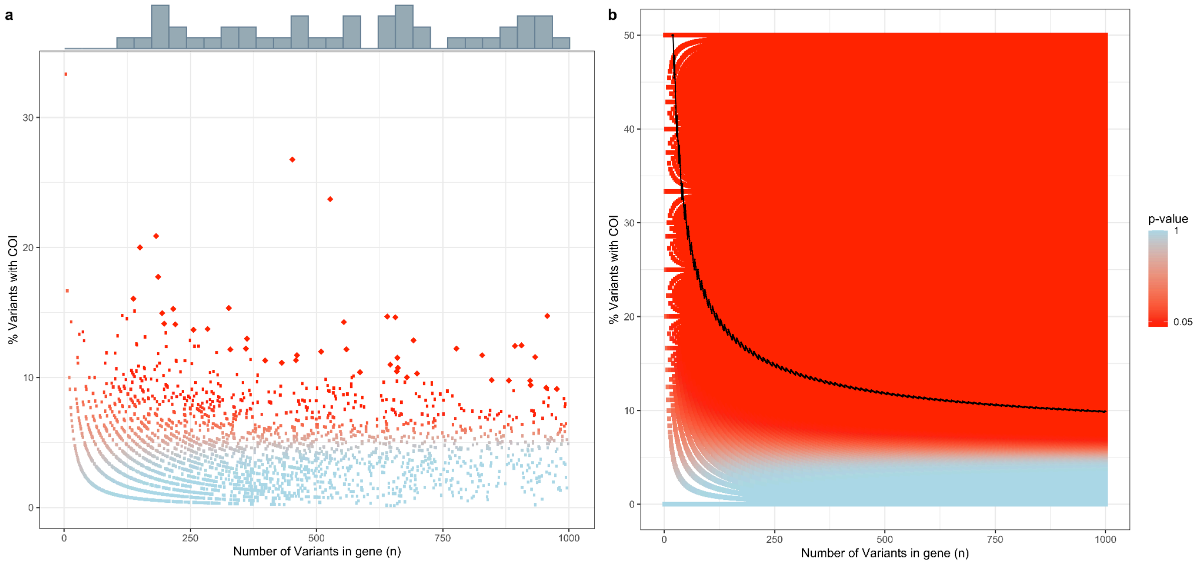

Supplementary Figure S7. The figure presents heatmaps displaying hypergeometric test p-values. These p-values are dependent on the number of variants in a gene (n) and the percentage of variants with conflicting interpretations of pathogenicity (COI). Data is shown for both real observations (a) and simulated scenarios encompassing all possible combinations of n and the percentage of variants with COI (b). The histogram on the top of (a) presents the distribution of genes based on n.
